# Nanoparticle-based materials in anticancer drug delivery: Current and future prospects

**DOI:** 10.1016/j.heliyon.2023.e21227

**Published:** 2023-10-20

**Authors:** Saniha Ajith, Fares Almomani, Abdelbary Elhissi, Ghaleb A. Husseini

**Affiliations:** a Department of Chemical Engineering, College of Engineering, Qatar University, Doha, Qatar; b College of Pharmacy, QU Health, Qatar University, Doha, Qatar; c Department of Chemical Engineering, College of Engineering, American University of Sharjah, United Arab Emirates; d Materials Science and Engineering Program, College of Arts and Sciences, American University of Sharjah, Sharjah, P.O. Box 26666, United Arab Emirates

**Keywords:** Drug delivery, Nanomaterials, Cancer therapy, Nanocarriers, Nanomedicine

## Abstract

The past decade has witnessed a breakthrough in novel strategies to treat cancer. One of the most common cancer treatment modalities is chemotherapy which involves administering anti-cancer drugs to the body. However, these drugs can lead to undesirable side effects on healthy cells. To overcome this challenge and improve cancer cell targeting, many novel nanocarriers have been developed to deliver drugs directly to the cancerous cells and minimize effects on the healthy tissues. The majority of the research studies conclude that using drugs encapsulated in nanocarriers is a much safer and more effective alternative than delivering the drug alone in its free form. This review provides a summary of the types of nanocarriers mainly studied for cancer drug delivery, namely: liposomes, polymeric micelles, dendrimers, magnetic nanoparticles, mesoporous nanoparticles, gold nanoparticles, carbon nanotubes and quantum dots. In this review, the synthesis, applications, advantages, disadvantages, and previous studies of these nanomaterials are discussed in detail. Furthermore, the future opportunities and possible challenges of translating these materials into clinical applications are also reported.

## Abbreviations

AgNP: Silver nanoparticles
AuNP: Gold nanoparticles
CMC: Critical micellar concentration
CNT: Carbon nanotubes
CUR: Curcumin
DDS: Drug delivery system
DMPC: Dimyristoyl phosphatidylcholine
DOX: Doxorubicin hydrochloride
DPPC: Dipalmitoyl phosphatidylcholine
EMA: European Medicines Agency
EPC: Egg phosphatidylcholine
EPR: Enhanced permeability and retention
ER: Estrogen receptors
FDA: Food and Drug Administration
GPCR: G-protein coupled receptors
HER-2: human epidermal growth factor receptors 2
LSPR: Localized surface plasmon resonance
MNP: Magnetic nanoparticles
MRI: Magnetic resonance imaging
MSNP: Mesoporous silica nanoparticles
MWCNT: Multi-wall carbon nanotubes
PECVD: Plasma-enhanced chemical vapor deposition
PEG: Polyethylene glycol
PEO: Polyethylene oxide
PET: Positron emission tomography
PTX: Paclitaxel
QD: Quantum dots
RES: Reticuloendothelial system
SEM: Scanning Electron Microscope
SPC: Soya phosphatidylcholine
SPION: Superparamagnetic iron oxide nanoparticles
SWCNT: Single-wall carbon nanotubes
TEM: Transmission Electron Microscope

## 1 Introduction

In the United States in 2022 alone, the American Cancer Society predicts 1,918,030 new cancer cases and 609,360 related deaths [1]. The likelihood of cancer becoming the leading cause of death by 2030 is anticipated to increase in the following years [2]. According to data published by the National Cancer Institute, the most prevalent types of cancer in males include stomach, liver and prostate, whereas most common types of cancers in females include thyroid, breast and cervical; by contrast, lung and colorectal cancers are among the most common in both genders [3]. Some common triggers of cancer include tobacco smoke, radiation, UV rays, etc.; however, genetics play a major role in determining a person's susceptibility to develop the disease. Typical carcinogens include benzene, beryllium, asbestos, formaldehyde, vinyl chloride, and arsenic [4].

Apoptosis or programmed cell death is a natural homeostatic mechanism to maintain and control cell populations. A change or damage to cellular DNA causes it to die, commonly known as ‘cellular suicide.’ It can also function as a defense mechanism when cells are damaged. In certain cases, when cells cannot undergo apoptosis, uncontrolled cell growth and division occur, leading to development of cancerous cells. Cancer cells are predatory, hence they can form blood vessels from nearby healthy tissues to supply the growing tumor with nutrients and oxygen [5]. The most typical treatment modalities include chemotherapy, surgery, immunotherapy, and radiation therapy.

In chemotherapy, anti-neoplastic agents are injected into the body to slow down the growth of cancer cells, or to kill them. 5-fluorouracil (5-FU)3 phenylalanine mustard, discovered by Heidelberger, was the first successful chemotherapy drug designed for melanoma in 1983 [6]. A major limitation of chemotherapy is that the drug does not properly distinguish between cancerous cells and normal cells. Therefore, these drugs can target and kill fast-growing normal cells in the body, leading to various adverse effects including fatigue, nausea, hair fall, infection, mucositis, and diarrhea. Moreover, chemotherapeutic drugs are strongly linked with cardiotoxicity and lowered immune functions [7].

In the progressive journey towards the achievement of modern personalized medicine, a relatively new scientific field has emerged, namely the field of ‘nanomedicine’ which relies on employing nanoparticles for diagnosis, imaging, monitoring, and treatment of diseases [8]. It is believed that nanoparticles in the size range of 10–100 nm are greatly advantageous for drug delivery applications. If smaller than 10 nm, they would be rapidly cleared from the bloodstream via the kidneys, and if greater than 100 nm, they may be quickly identified and eliminated by the reticuloendothelial system (RES); thus for appropriate cancer targeting an appropriate size range is essential [8]. Nanoparticles have also been widely studied in central nervous system (CNS) drug delivery. CNS disorders such as Alzheimer's disease, Huntington's disease, and frontotemporal dementia are on the rise. A major challenge of treating CNS disorders is the presence of the blood-brain barrier (BBB) and cerebrospinal fluid, which hinders drugs from reaching the targeted cells [9]. Hence, in recent years, more effort has been directed toward studying how nanotechnology-based systems can be used for delivering drugs to the brain and spinal cord [10].

Nanomaterials for drug delivery are of two types: organic and inorganic. Organic nanocarriers like liposomes, dendrimers, and micelles are highly biocompatible and biodegradable. However, they generally have low drug-loading capacities and reduced stability. By contrast, inorganic nanocarriers like metallic and carbon nanoparticles are more stable with multifunctional capabilities but are less biocompatible and biodegradable. Table 1 briefly summarizes the advantages and disadvantages of both, organic and inorganic nanocarriers.

**Table 1:** Advantages and disadvantages of organic and inorganic nanocarriers.

Type of carrier	Advantages	Disadvantages	References
Organic nanocarriers			
Liposomes	- Amphiphilic - Maintains structure at higher temperatures - Highly biocompatible and biodegradable - Excellent cell internalization mechanism	- Low solubility in blood - Shorter half-lives - Low drug loading efficiency	[11,12]
Polymeric micelles	- Does not dissociate rapidly due to high molecular weight - High stability - Longer shelf life - Inexpensive and easy to synthesize	- Potential toxicity - Low drug loading efficiency	[13,14]
Dendrimers	- Polyvalency allows multifunctional surface conjugation - Increased surface area	- Scattered hyperbranched structure - Complex synthesis techniques - Unsuitable for incorporation of hydrophilic drugs	[15,16]
Inorganic nanocarriers			
Magnetic nanoparticles	- Easy elimination from the body through metabolic iron pathways - Mechanical and chemical stability - Uniformity in size - Use as MRI contrast agents	- Potential toxicity - Challenge to direct MNPs to desired tissue in the absence of magnetic field	[17,18]
Mesoporous silica nanoparticles	- High stability - Large surface area - Excellent loading capacity	- Scattered size distribution - Potential toxicity	[19,20]
Gold nanoparticles	- Suitable for photodynamic therapy - Large surface area - High stability	- Expensive for commercial production - Non-biodegradable	[21,22]
Carbon nanotubes	- Rigid and sturdy - High electrical conductivity - Water dispersible	- Particle aggregation - Potential toxicity (not biodegradable) and low biocompatibility	[23,24]
Quantum dots	- High penetration capacity in biological media - Excellent optical properties	- Cannot dissolve in solution easily - Toxicity	[25,26]

**Fig. 1:**
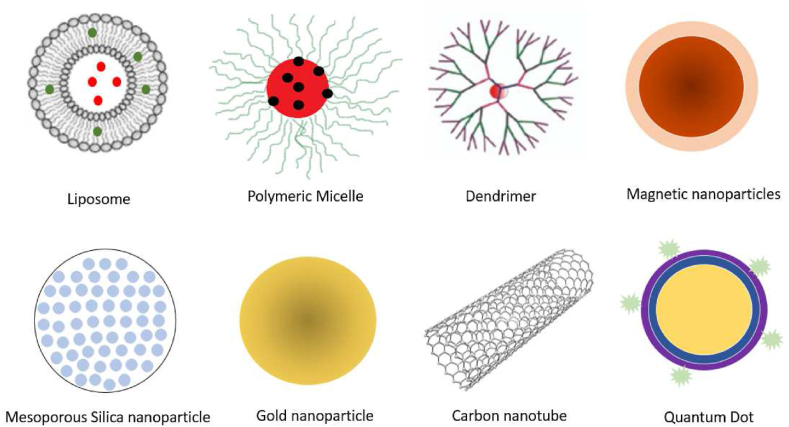
Nano-systems used in cancer drug delivery.

In this review, the use of nanoparticles for cancer drug delivery and targeting was explored. The formulation, basic elements, and method of delivery were highlighted and discussed. In this report, we have reviewed a broad range of nanoparticles used in anticancer drug delivery (as seen in Fig. 1), including liposomes, polymeric micelles, dendrimers, magnetic nanoparticles (MNPs), gold nanoparticles (AuNPs), carbon nanotubes (CNTs), mesoporous nanoparticles (MSNPs), and quantum dots (QD). Several other nanoparticles such as solid lipid nanoparticles (SLN), silver nanoparticles (AgNPs), nanowafers and nanorods have also been studied for their use in anticancer drug delivery; however, due to limited number of studies, they have not been discussed in detail in this review.

## 2 Methodology

This review provides a summary of the literature on cancer drug delivery published between 2015 and 2023. A handful of articles from earlier years have also been included as they contain important data about the history and development of nanomedicine. The primary source of these articles was procured from Google Scholar, ScienceDirect, PubMed and Scopus databases; the keywords applied for the search were “drug delivery,” “nanocarriers,” “cancer drug delivery,” “tumor drug delivery,” and “nanomedicine.” The collected data were discussed in detail in 15 different sections. Sections 1, 2, 3, 4, 5 provided a general introduction to the topic. Section 1 discusses the pathology of cancer, the importance of cancer treatment, statistics of cancer diagnosis worldwide, drawbacks of conventional treatments and the necessity to introduce drug delivery systems for effective treatment. Section 3 introduces the concept of smart drug delivery systems and stimuli-sensitive nanoparticles. Section 4 discusses the important concept of the ‘enhanced permeability and retention (EPR)’ effect, which is the principal mechanism through which nanomaterials accumulate at the cancer site. The use of polyethylene glycol (PEG) as a coating for nanocarriers to avoid opsonization by the immune system is explained in Section 5.

Sections 6, 7, 8, 9, 10, 11, 12, 13 discuss eight major nanocarriers used in drug delivery. These include liposomes (Section 6), polymeric micelles (Section 7), dendrimers (Section 8), magnetic nanoparticles (Section 9), mesoporous silica nanoparticles (Section 10), gold nanoparticles (Section 11), carbon nanotubes (Section 12) and quantum dots (Section 13). Their synthesis, applications, advantages, and disadvantages have been investigated in detail. Additionally, critical analyses of various *in vitro* and *in vivo* studies found in literature have also been included in these sections. Finally, sections 14, 15 discuss the future and challenges of cancer drug delivery, along with concluding remarks.

## 3 Smart drug delivery systems

Drug delivery systems (DDS) include formulations, technologies, and devices used to deliver pharmaceutical and medical products required by the body in order to achieve the desired therapeutic effect safely. Conventional drug delivery systems utilize anatomical pathways such as oral, inhalation, transmucosal, or transdermal to deliver drugs to diseased tissues. However, in recent years, these DDS have evolved to become ‘smarter’ with stimuli-responsive qualities. These smart drug delivery systems include a drug encapsulated nanocarrier which can respond to either exogenous or endogenous triggers to release the drug or enhance its targetability [27].

Exogenous triggers are external triggers that are applied externally to enhance drug release. These include ultrasound, magnetic field, temperature, electricity, or light. In contrast, endogenous triggers are internal triggers that are related to the physio-chemical characteristics of the disease, such as pH differences, enzyme concentrations, redox gradient, or hormone variations. Table 2, Table 3 summarize the advantages and disadvantages of some exogenous and endogenous triggers, respectively.

**Table 2:** Advantages and disadvantages of exogenous triggers.

Type of exogenous trigger	Advantages	Disadvantages	References
Light (visible/NIR)	- High precision - No harmful radiation - Inexpensive	- Poor tissue penetration	[28,29]
Magnetic field	- No ionizing radiation - Can be used in MRI applications	- Potential toxicity from iron oxide - Expensive	[8,30]
Ultrasound	- Inexpensive - Safe to use - Non-invasive	- Increase in physiological temperature - Risk of tissue damage at high intensity	[22,31]
Temperature	- Cancer cells are sensitive to increases in temperatures - Wide range of applications	- Risk of tissue damage from elevated temperature	[32,33]

**Table 3:** Advantages and disadvantages of endogenous triggers.

Type of endogenous trigger	Advantages	Disadvantages	References
pH	- Minimal pH change is enough to trigger drug release	- Low accuracy - Off-target delivery due to pH gradient within the tumor	[22,33]
Redox	- Extremely sensitive - Highly stable in biological tissues - Quick reaction to high GSH concentration in cancer cells	- Off-target delivery	[34]
Enzymatic	- Excellent control over spatiotemporal drug release	- Challenge of enzyme dysregulation in different tumors - Need for specific enzymatic drug delivery design	[33,35]

## 4 Enhanced permeability and retention effect

**Fig. 2:**
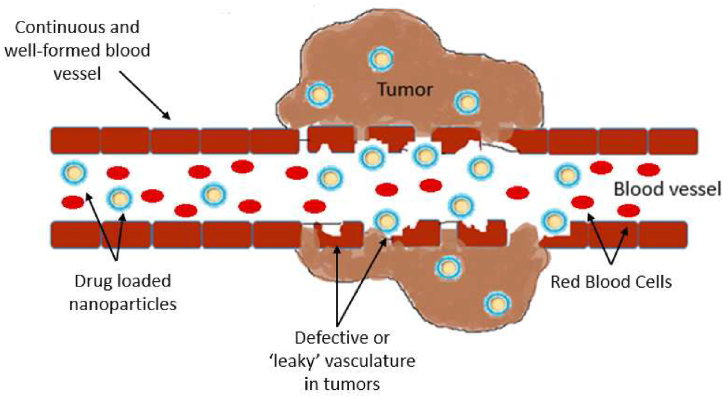
Enhanced permeability and retention (EPR) effect.

Matsumura and Maeda initially proposed the enhanced permeability and retention (EPR) effect in 1986 [36]. Their study revealed that tumor-damaged blood vessels exhibit increased vascular permeability. These leaky vasculatures enable nanomaterials in the size range of 10–150 nm to reach the tumor site easily, with exhibited affinity to be retained within the tumor, owing to the positive pressure from blood vesicles to the tumor site. Thus, formulation scientists should take advantage of the EPR effect when designing intravenous anticancer drug delivery systems by manufacturing the anticancer nanomedicine in this advantageous size range [37]. Fig. 2 shows the difference between the damaged blood vessel near the tumor site and the continuous, well-formed blood vessel near the healthy tissue. The defective or leaky vasculature provides gaps for the drug-loaded nanoparticles to enter the tumor site. This abnormal tumor vasculature characteristics is highly advantageous for delivering chemotherapy drugs directly to the cancer cells while minimizing the effect on the healthy cells.

### 4.1 Active and passive targeting

Nanoparticles enter cells via a number of reported mechanisms; a prominent one is receptor-mediated endocytosis [38]. To maximize the cellular uptake of these nanocarriers, ‘active targeting’ strategies can be used. In active targeting, specific molecules such as peptides, antibodies, proteins, or other small molecules are attached to the surface of drug-loaded nanocarriers via conjugation chemistry [39]. Cancer cells overexpress specific receptors on their surface. For example, G-protein coupled receptors (GPCR) are overexpressed on lung cancer cells [40], while estrogen (ER) receptors, progesterone (PR) receptors or human epidermal growth factor receptors-2 (HER2) are overexpressed on breast cancer cells [41]. The conjugated molecules on the nanoparticles bind to the receptors on the surface of the cancer cells via ligand-receptor interactions, commonly referred to as the ‘key and lock’ mechanism [42]. In contrast to normal, healthy tissues, tumour tissues present a broken and poorly arranged vasculature and an absence of a lymphatic system which prevents the clearance of internalized particles. A nanocarrier loaded with the chemotherapeutic drug can easily enter via this vasculature via ‘passive targeting’ mechanisms.

## 5 PEGylated nanomaterials for cancer treatment

A major drawback of using nanocarriers is their quick opsonization and destruction by the reticuloendothelial system (RES). Opsonin proteins circulating in the bloodstream can adsorb on the surface of the nanocarriers, resulting in subsequent elimination by macrophages. This issue can be overcome by coating the nanocarrier with hydrophilic polymers such as polyethylene glycol (PEG), which is known to influence the pharmacokinetics of the carrier. PEG is a linear polymer with many useful characteristics, such as biocompatibility, excellent solubility, low toxicity, excellent bioelimination, and low immunogenicity [43]. PEG incorporation on nanocarrier surfaces (i.e. PEGylation) can significantly increase the circulation time of the nanoparticles in the blood stream, making it easier for the carriers to reach the targeted site without being attacked by the immune system; this observation is commonly described as “passive targeting”, and was first introduced in the late 1970's [44].

## 6 Liposomes

Liposomes are spherical vesicles composed of single or multiple lipid bilayers, which isolate their aqueous interior from the external medium. They are the result of an accidental discovery by the British biophysicist, Dr. Alec D Bingham in 1960s. He discovered that a closed bilayer structure was formed when water was added to the phospholipid phosphatidylcholine [45]. Liposomes comprise phospholipids that can be produced synthetically or extracted from nature (e.g. soybean, egg yolk, etc.). Examples of natural phospholipids include egg phosphatidylcholine (EPC) and soya phosphatidylcholine (SPC); whereas synthetic phospholipids include dimyristoyl phosphatidylcholine (DMPC) and dipalmitoyl phosphatidylcholine (DPPC). Each Phospholipid molecule has two hydrophobic tails and a hydrophilic headgroup comprising choline, ethanolamine, inositol or serine [46]. The “phase transition temperature” is an essential property of phospholipid molecules, and hence, liposomes. It is defined as the temperature needed to rearrange the ordered gel phase of a lipid to its disordered liquid phase. Factors such as the length of hydrocarbon, saturation, charge, and headgroup can affect the phase transition temperature [46].

**Fig. 3:**
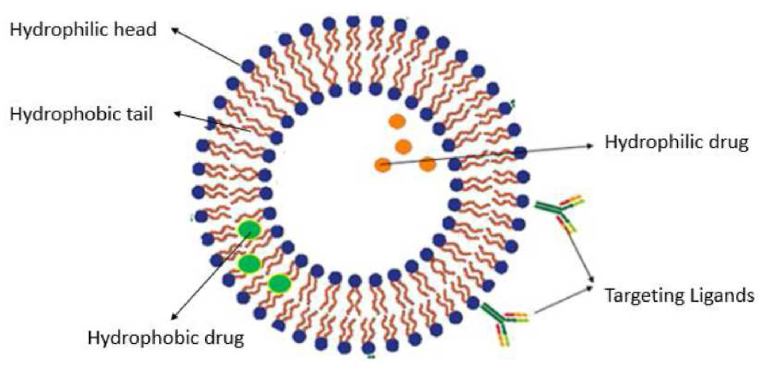
Structure of a liposome. Reprinted with permission from Ref. [12], S. Ajith, “A novel cancer treatment platform utilizing HER2-immunoliposomes and ultrasound,” Dept. Chem. Eng., American University of Sharjah, Sharjah, UAE, **2020**.

The unique structure of liposomes allows them to carry a variety of bioactive molecules such as genes, viruses, drugs, DNA, proteins, vaccines, and enzymes. Additionally, they can also be used for molecular imaging due to their ability to encapsulate radioactive molecules [47]. Fig. 3 depicts the structure of a liposome [12].

Currently, several approved liposomal cancer treatments are commercially available, including Doxil®, Depocyt®, and Marqibo® [11]. Several other formulations have undergone clinical trials to assess their efficiency in cancer treatment. The clinical trials data of these liposomal formulations (as of 2023) are displayed in Table 4.

**Table 4:** Liposomal formulations for the treatment of various cancers (as of 2023).

Name of liposomal product	Name of drug	Type of cancer	Clinical Trial Phase	References
Myocet	Doxorubicin	Metastatic breast cancer	Approved by FDA	[48]
Doxil	Doxorubicin	Ovarian and breast cancer, Kaposi's sarcoma	Approved by FDA	[49]
DanuXome	Daunorubicin	Kaposi's sarcoma, Myeloid leukaemia	Approved byFDA	[50]
Marqibo	Vincristine sulphate	Acute lymphoblastic leukaemia	Approved by FDA	[51]
Alocrest	Vinorelbine	Breast cancer, solid tumors	Phase I	[52]
ThermoDox	Doxorubicin	Hepatocellular carcinoma	Phase III	[53]
EndoTAG-1	Paclitaxel	Metastatic pancreatic cancer, solid tumors	Phase II	[54]
Lipoplatin	Cisplatin	Pancreatic, breast, gastric, non-small cell lung cancer	Phase III	[55]
LEM-ETU	Mitoxantrone	Ovarian, breast, stomach cancers, and leukaemia	Phase I	[56]
CPX-1	Irinotecan HCL and floxuridine	Advanced Colorectal Cancer	Phase II	[57]

### 6.1 Methods of liposome formulation

Various methods to prepare liposomes have been described in literature. Among those, the most popular ones include thin-film hydration, reverse-phase evaporation, solvent-injection techniques, and detergent dialysis [58].

For the thin-film hydration technique, a vacuum is applied to an aqueous lipid solution within a round-bottom flask attached to a rotary evaporator to obtain a thin-lipid film on the flask. This is followed by the addition of the drug. Hydrophilic drugs accumulate into the aqueous phase, while hydrophobic drugs are embedded within the lipid phase. The final steps involve sonication and extrusion to reduce the size of liposomes [12].

The reverse-phase evaporation method commences by dissolving the lipid in an organic solvent. The drug to be encapsulated is added to it at freezing temperatures in a sonicating bath. A viscous gel is obtained by eliminating the solvent at low pressure. Liposomes are then formed by vigorously mixing the gel [59].

In the ethanol injection technique, the phospholipids must be dissolved in ethanol and injected into an aqueous solution while being constantly stirred. The biological molecule is dissolved in an inorganic solution, and the ethanol is evaporated under vacuum. The detergent dialysis technique follows a similar method as the thin-film hydration method. Once the thin film is formed, they are hydrated with an aqueous drug solution and freeze-dried using sodium chloride [59].

The above techniques limit the formulation of liposomes to lab-based settings. Commercialization and bulk manufacturing of liposomal drug formulations can be energy intensive and expensive. Additionally, long term storage of liquid liposome solutions can affect its stability and reduce its shelf-life. To overcome these challenges, Payne and co-workers introduced the concept of ‘proliposomes’ in 1986 [60]. They prepared sorbitol based proliposomes using egg lecithin, ergosterol and amphotericin B. Proliposomes are solid, granular particles which disperses to form an isotonic liposomal suspension when hydrated. It is thought that this proliposome system is ideal for generating liposomes that incorporate hydrophobic drugs where the drug can easily be encapsulated into the lipid bilayers of the liposomes. The amount of drug encapsulated depends on various factors such as lipid composition and hydration protocol [60].

Another approach to manufacturing proliposomes was introduced by Perrett et al. who prepared an ethanol-based proliposome formulation using egg phosphatidylcholine and cholesterol dissolved in alcohol. The addition of aqueous phase with shaking generates liposomes [61]. Results indicated excellent drug entrapment efficiency for hydrophilic drugs, and high stability upon long term storage. This method is highly applicable for large scale production of liposomes [61].

### 6.2 Advantages and disadvantages of liposomes

Several advantages exist for the use of liposomes in drug delivery. Firstly, they can be used to load hydrophilic, hydrophobic, and amphipathic molecules. The aqueous core accommodates hydrophilic entities, and the lipid membrane entraps hydrophobic molecules, while amphiphilic materials may partition between the aqueous core and the lipid membranes. Secondly, their size can be manipulated for various conditions, and the surface can be altered with multifunctional ligands. Lastly, their structural and compositional similarity to cell membranes makes them highly biocompatible and biodegradable; thus liposomes are known to be the safest nanocarrier systems [11].

Nevertheless, some limitations exist in using liposomes as drug delivery systems. Their low solubility in blood and short half-lives mean they cannot stay stable for long periods. The phospholipid could undergo hydrolysis and oxidation reactions, resulting in the possible leakage of the entrapped material during storage. In addition, the manufacturing process is expensive and, most methods used for manufacturing liposomes are employed at a small scale, and are not feasible for large-scale production. However, Gala et al. developed a novel method for large-scale liposome production using a triple approach. Hydrogenated soya phosphatidylcholine and sucrose were first used to synthesize proliposomes, which were then coated using the fluidized bed technology, and using beclomethasone dipropionate as a model hydrophobic drug. This was followed by high pressure homogenization and freeze-drying to yield stable nano-carriers. This triple approach technology can be applied for large scale production of proliposomes [62].

### 6.3 Applications of liposomes in cancer drug delivery

Liposomes have been shown to improve drug penetration and decrease drug clearance from the bloodstream. In a study by Elamir et al., doxorubicin-liposomes were prepared by coating with the monoclonal antibody Trastuzumab to target the overexpressed HER2 receptors in breast cancer cells. Low-frequency ultrasound was then used to trigger cavitation in the liposomes, leading to the release of the drug. *In vitro* studies on the HER2+ cell line (SKBR3) showed that the Trastuzumab-liposomes exhibited higher cellular toxicity and facilitated better drug uptake in the cells compared to free liposomes. This demonstrated that combining ultrasound with immunoliposomes can be a promising strategy to improve the delivery of chemotherapeutics to cancer cells [63]. Ultrasound is a safe, minimally invasive trigger for drug delivery. However, most of the studies in literature only portray the safety of liposomes in combination with low-frequency ultrasound [[64], [65], [66], [67]]. High-frequency ultrasound (15–20 MHz) provides the same therapeutic benefits as that of the low-frequency one [68].

Photodynamic therapy is a novel approach to treat several types of skin cancer. It uses special photosensitizer molecules which when activated by light rays, can damage micro-cell structures in mammalian cells or microorganisms. Although this is effective in destroying cancer cells, several drawbacks such as minimal biocompatibility, coagulation and aggregation prevent the use of photodynamic therapy in a clinical setting. To overcome these challenges, liposomes loaded with photosensitizers were developed to improve stability. The photosensitizer molecules used in the study were acridine orange (AO) and methylene blue (MB). Results showed excellent stability of the molecules within the lipid-based liposomes which were further stabilized with cholesterol and sodium cholate surfactant. AO-encapsulated liposomes had an encapsulation efficiency of 98 %, while MB encapsulation efficiency was only 86 % [69].

Additionally, several studies have been conducted in the last few years for the use of coated liposomes like glucose [70], fucoidan [71], hyaluronic acid [72], transferrin [73], folic acid [74], etc. Investigations on liposomal co-delivery of chemotherapeutic drugs such as docetaxel and resveratrol [75], SN38 prodrug and curcumin [76], cisplatin and nitroxoline [77], gemcitabine and cisplatin [78], doxorubicin and lovastatin [79], etc have also been studied to induce a synergistic effect. Research has also been conducted to test the possibility of delivering chemotherapeutics with siRNA to improve synergistic therapeutic efficacy [80]. Table 5 summarizes recent *in vivo* studies utilizing liposomes as an anti-cancer agent carrier.

**Table 5:** Summary of *in vivo* studies using liposomes for cancer drug delivery.

Liposome type	Anti-cancer agent	Cell line for *in vivo* testing	Year study published	Reference
Alginate/chitosan/aptamer liposome	5-Fluorouracil	HT-29 colorectal adenocarcinoma cells (non-small cell lung cancer)	2022	[81]
Glycyrrhetinic acid/triphenylphosphine liposome	Doxorubicin	HepG2 hepatoma (liver cancer)	2022	[82]
Trastuzumab- liposome	Doxorubicin	SKBR3 and MDA-MB-231 (breast cancer)	2021	[63]
DMPC/DSPC cholesterol liposomes	Doxorubicin	ER+/PR+ and MCF-7 (breast cancer)	2021	[83]
Transferrin-gold liposomes	Docetaxel	C6 glioma cells	2021	[84]
DPPC- DSPE PEG liposomes	Doxorubicin	DU145 and PC3 (prostate cancer)	2020	[85]
DMPC/DMPC cholesterol liposomes	Curcumin	A2780 (ovarian cancer)	2019	[86]
Hyaluronic acid- liposomes	Doxorubicin and Paclitaxel	MCF-7 (breast cancer)	2019	[87]
DMPC/DSPC cholesterol liposomes	Sirolimus	LNCaP and DU145 (prostate cancer)	2021	[88]
Hyaluronic acid- liposomes	Shikonin	MDA-MB-231 (breast cancer)	2022	[89]
DMPC/DSPC cholesterol liposomes	Azadiradione	MDA-MB-231 (breast cancer)	2021	[90]
Transferrin-liposomes	Docetaxel	PC-3 and PNT2 (prostate cancer)	2021	[91]
DOPE and Soy liposomes	Docetaxel and Pemetrexed	MC-38 and CT-26 (colon cancer)	2023	[92]

## 7 Polymeric micelles

**Fig. 4:**
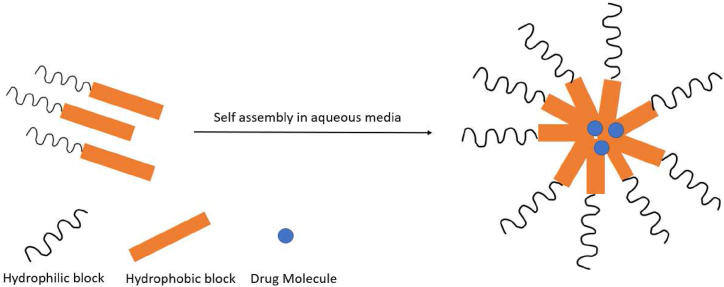
Drug-loaded polymeric micelle formed by self -assembly of amphiphilic block copolymers.

Polymeric micelles are an aggregate of colloids which are formed by the self-assembly of amphiphilic block copolymers in aqueous media [93]. These micelles have a hydrophilic outer shell and hydrophobic inner core, as portrayed in Fig. 4. Bioactive hydrophobic molecules can be loaded into the core, while the hydrophilic corona shell protects the core and provides compatibility within the aqueous system. The ‘critical micellar concentration’ (CMC) is an important characteristic of micelles, which is the lowest concentration needed for the surfactant molecules to aggregate and assemble themselves in a micellar shape. At low CMC levels, the copolymers remain dispersed in the bulk solution [94].

The most common copolymer used is poly (ethylene glycol) (PEG). Other common block copolymers are poly (N-isopropylacrylamide) (PNIPAAM), poly (N-vinyl pyrrolidone) (PVP), and chitosan. Poly (propylene oxide) (PPO), poly(L-lactide) (PLA), poly-ƹ -caprolactone (PCL), poly (lactide-co-glycolic acid) (PLGA) and poly (β-amino esters) are also widely used. A commercially available polymeric micelle formulation for drug delivery is Pluronic®, which is composed of triblock copolymers – poly (ethylene oxide) and poly (propylene oxide) [95].

### 7.1 Advantages and disadvantages of polymeric micelles

Several advantages of polymeric micelles over other nanocarriers have been reported. The high molecular weight of these molecules ensures that they do not dissociate instantaneously upon dilution. As a result, they are considerably more stable compared to micelles of lower molecular weights. They are also highly stable in the bloodstream and can circulate for prolonged periods [13]. Additionally, they have a longer shelf life upon storage. The typical size of polymeric micelles is around 15–30 nm, which means they can escape renal excretion and accumulate at the tumor site. Small molecules and drugs can easily be loaded into these micellar structures using simple techniques. Furthermore, most polymers used to synthesize micelles are inexpensive and have low toxicity [96].

However, a significant drawback of polymeric micelles is their rapid clearance from the bloodstream. This can be overcome by incorporating an exterior layer of polyethylene oxide (PEO)/polyethylene glycol (PEG) on the micellar structure. The hydrophilic nature of PEG causes it to bind with water molecules, which leads to a lowered protein adsorption on the micellar surface. This means they can travel in the bloodstream for prolonged periods and avoid phagocytosis [97].

### 7.2 Applications of polymeric micelles in cancer drug delivery

Polymeric micelles are popular nanocarriers for drug delivery due to their ease of tissue penetration without being recognized by the immune system. They also exhibit excellent drug loading efficiency and biodegradability. In some cases, polymeric micelles could be destroyed by digestive enzymes if administered orally. Many studies have been conducted to test the efficiency of targeting cancer cells with the help of polymeric micelles.

Xiang et al. used polymeric micellar drug delivery systems as an effective breast cancer treatment platform. They fabricated a polymeric micelle from poly (ethylene glycol)-*block*-dendritic polylysine (PEITC) and encapsulated the chemotherapeutic drug Paclitaxel (PTX), inside its core. Incorporating PTX into the polymeric micelles could potentially improve its solubility and enhance its selectivity toward cancerous cells. *In vivo* research was conducted where the efficiency of the synthesized PTX-loaded PEITC was compared to PTX-loaded poly (ethylene glycol)-*block*-poly (*D*, *L*-lactide), a clinically available micellar formulation known as Genexol®. Experiments on subcutaneous and human breast cancer xenografts revealed increased tumour accumulation, enhanced therapeutic efficiency, and lower blood clearance [98].

Other studies show the efficiency of chitosan-based camptothecin micelles for the effective treatment of colorectal cancer [99]. Micelles have also been effectively used in photodynamic therapy for breast cancer by encapsulating hypericin [100]. More interestingly, researchers have also tried to combine micelles with other nanoparticles to deliver drugs such as the polymeric micelle capped mesoporous silica nanoparticle for thermo responsive dual drug delivery [101]. Table 6 outlines the recent studies of polymeric micelles used as nanocarriers in cancer therapy.

**Table 6:** Summary of *in vivo* studies using polymeric micelles for cancer drug delivery.

Polymeric Micelle type	Anti-cancer agent	Cell line for *in vivo* testing	Year study published	Reference
PEGylated N-(2 hydroxypropyl) methacrylamide polymeric micelles	Doxorubicin	MDA-MB-231 (breast cancer)	2021	[102]
Degradable polycarbonate polymeric micelle	Doxorubicin	CT26 (colon cancer)	2021	[103]
β-CD-(PCL-PAEMA)21 – aptamer polymeric micelle	Camptothecin	4T1 and MCF-7 (breast cancer)	2022	[104]
PEG- cholesterol polymeric micelle	Cabazitaxel	C4-2 (prostate cancer)	2020	[35]
Folated PEG-PAA polymeric micelle	Dactolisib	KB and A549 (lung cancer)	2020	[105]
Coumarin and imidazole grafted PEG-PLL polymeric micelle	Doxorubicin	4T1 (breast cancer)	2020	[106]
Pluronic F127 polymeric micelle	Zileuton™	MCF-7 and MDA-MB-231 (breast cancer)	2020	[107]
PEG- resorcinol polymeric micelle	Doxorubicin	MCF-7 (breast cancer)	2023	[108]
RGD PEG- PTX polymeric micelle	Paclitaxel	SGC7901 (gastric cancer)	2019	[109]
P(MeOx-b-BuOx-b-MeOx polymeric micelle	Paclitaxel and Cisplatin co-delivery	A2780 and LCC-6-MDR (ovarian cancer)	2019	[110]

## 8 Dendrimers

**Fig. 5:**
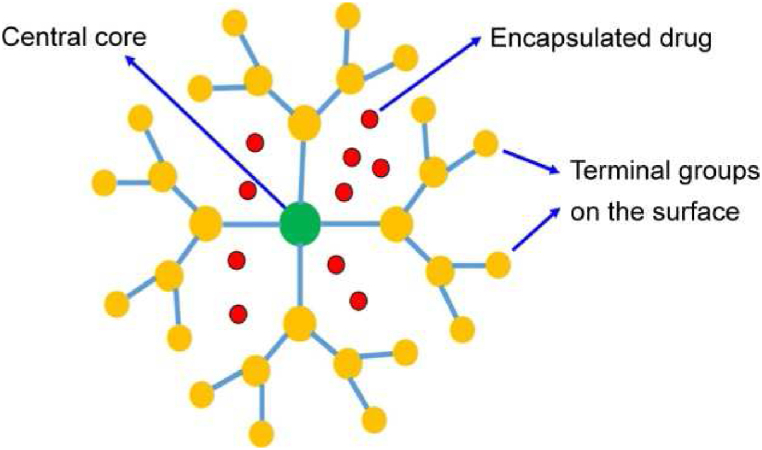
Structure of dendrimer with the encapsulated drug. Reprinted with permission from Ref. [111], Din et al., “Effective use of nanocarriers as drug delivery systems for the treatment of selected tumors,” *Int J Nanomed.*, vol. 12, pp. 7291–7309, **2017**.

Dendrimers are a special class of globular polymeric nanocarriers with a unique hyperbranched structure, different from the conventional linear polymeric structures. They are homogenous and monodisperse molecules consisting of tree-like branches, as seen in Fig. 5 [111]. They were first discovered in 1978 by Fritz Vogtle. Dendrimers have three distinguishing features in their structure: i) a core, ii) repeating units originating from the core, and iii) an exterior layer region [112]. The first dendrimer synthesized in the lab was polyamidoamine (PAMAM), which uses ammonia as the core molecule [113].

Several types of dendrimers have been discovered in the past years. These dendrimers, along with their key features, have been portrayed in Table 7 [113].

**Table 7:** Types of dendrimers and their key features [113].

Types of dendrimers	Key Features
Polyamidoamine (PAMAM) dendrimers	- Synthesized using ammonia as core - Commercially available under the trademark name ‘Starburst’
Polyamidoamine-organosilicon (PAMAMOS) dendrimers	- Consist of hydrophilic PAMAM core and hydrophobic organosilicon exterior
Polypropylene imine (PPI) dendrimers	- Commercially used in materials science and biology fields - Also referred to as ‘DAB-dendrimers’ where DAB stands for diamino butane core
Tecto dendrimers	- Consist of core dendrimer and several exterior dendrimers - Used for various applications like drug delivery, diagnosis, and cell recognition
Multilingual dendrimers	- Multiple copies of the same functional group exist on the dendrimer surface
Amphiphilic dendrimers	- Consist of two electronically opposite sides; one side is electron donating while the other is electron receiving
Hybrid dendrimers	- Hybrids of dendritic and linear polymers
Frechet-type dendrimers	- Novel class of dendrimers - Carboxylic acid groups on the surface for functional groups - Based on poly benzyl ether

### 8.1 Methods of dendrimer synthesis

One of the earliest techniques employed to manufacture dendrimers is the ‘divergent methodology’ introduced by D. Tomalia in the 1980's [114]. In this method, reactive monomeric molecules are coupled with the atoms making up the core. An example is the PAMAM-NH_2_ dendrimers which were synthesized by linking ammonia to N-(2-aminoethyl) acryl amide monomers. The greatest advantage of this method is that dendrimers can be modified by altering terminal groups to fit the required application. However, a limitation of this approach is that structural defects in the dendrimer can occur due to incomplete reactions, rendering them unfit to load therapeutic agents or diagnostic tools [115].

In the 1990's, Frechet and Hawker introduced the ‘convergent method’ to synthesize dendrimers. This technique produces dendrimers of uniform homogeneity and precise molecular weights. Reactive monomers are slowly assembled on the surface units to produce nanoparticles. A disadvantage of this technique is that it produces a low yield and is suitable only for low-generation dendrimer production [116].

Other methods involve the ‘double exponential’ method and ‘hyper core and branched monomers growth’ method. The former technique uses a single group for the convergent and divergent growth of the monomer unit, while the latter employs oligomeric species to produce dendrimers at a high yield [112]. Yet another novel synthesis technique is the ‘lego chemistry’ approach first demonstrated by Tomalia and Svenson, which uses branched monomers and a functionalized core to produce phosphorous dendrimers [117]. Currently, extensive research is being conducted to find novel and cost-effective strategies to produce dendrimers at commercial scales.

### 8.2 Advantages and disadvantages of dendrimers

A great benefit of dendrimers is that they are monodisperse, unlike linear polymers. They are often referred to as ‘artificial proteins’ because of their biomimetic properties and narrow size ranges. However, dendrimers are less compact than proteins due to their hyperbranched structure. The highly polyvalent surface of dendrimers allows for the conjugation of a significant number of functional groups [118]. Several dendrimers with different properties have been synthesized for various applications in recent years.

### 8.3 Applications of dendrimers in anticancer drug delivery

**Fig. 6:**
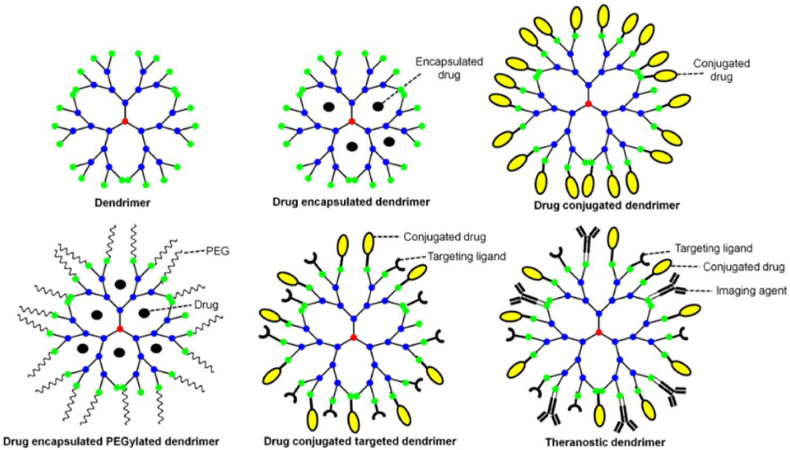
Types of theragnostic dendrimers. Reprinted with permission from Ref. [120], Saluja et al., “Dendrimers based cancer nanotheranostics: An overview,” *Int*. *J*. *Pharm*., vol. 600, pp. 120485, **2021**.

In the past decade, nanomedicine has seen a rise in the use of dendrimers due to their desirable features such as high water solubility, precise molecular weight, multivalency and biocompatibility [119]. Anti-cancer drugs can either be encapsulated within or conjugated to the surface of dendrimers. In some cases, PEG or other targeting ligands can be added to protect the dendrimer from opsonization and to ensure the active targeting of the cancer cells. A schematic of various types of theragnostic dendrimers has been portrayed by Saluja et al., and is displayed in Fig. 6 [120]. One of the earliest synthesized and commonly used dendrimers is poly(amidoamine) or PAMAM dendrimers. These are well-defined, biocompatible and have a small polydispersity index. PAMAM dendrimers also offer the advantage of surface functionalities and have been widely studied as a potential gene vector and drug carrier [121].

Fluorinated PAMAM dendrimers encapsulated with trastuzumab have been tested for the treatment of HER-2-positive breast cancer. This drug delivery system is unique with the addition of fluorine which can be used to track treatment via MRI measurements [122]. Very recently, Mignani et al. have claimed the importance and benefits of phosphorus dendrimers. When a phosphorus group exists in any position in the dendrimer, its properties are enhanced which makes them an excellent candidate for diagnosis, imaging, and drug delivery. Moreover, the research also claims that phosphorous dendrimers alone can be used as therapeutics for a wide range of diseases such as cancer, inflammations and neurodegenerative diseases, albeit a lack of studies in this area exists [123].

Dendrimers have also been investigated for the co-delivery of chemotherapeutics and/or siRNA to enhance synergistic effect for cancer therapy. One example of co-delivery is the conjugation of both doxorubicin and cisplatin in hyaluronic acid modified PAMAM dendrimers [124]. Aptamers, which are single stranded RNA or DNA molecules, have also been investigated as a platform for dendrimer drug delivery. Aptamers are known to improve ligand binding capacity, while maintaining stability and increasing drug loading efficiency [125]. Table 8 outlines the recent studies of dendrimers used as nanocarriers in cancer therapy.

**Table 8:** Summary of *in vivo* studies using dendrimers for cancer drug delivery.

Dendrimer type	Anti-cancer agent	Cell line for *in vivo* testing	Year study published	Reference
PAMAM G3	Lapatinib and fulvestrant	MCF-7, MDA-MB-231 and HER2- positive (breast cancer)	2022	[126]
Glycosylated PAMAM	Methotrexate	MDA-MB-231 (breast cancer)	2020	[127]
PAMAM	Celastrol	SW620	2020	[128]
Fluorinated PAMAM G5	Trastuzumab	MCF-7 (breast cancer)	2021	[122]
Hyaluronic acid-PAMAM	Doxorubicin and Cisplatin co-delivery	MCF-7 and MDA-MB-231 (breast cancer)	2019	[124]
Graphene oxide – triazine	Doxorubicin	MCF-10 A normal cells and MCF-7 (breast cancer)	2021	[129]
PEGylated PAMAM	Bortezomib	A549 (lung cancer) and MCF-7 (breast cancer)	2020	[130]
Peptide dendrimer	Doxorubicin	Pancreatic cancer cells	2022	[131]
PAMAM - D	Vismodegib	HaCaT (skin cancer)	2022	[132]
PLA and HA-modified PAMAM G4.5	Paclitaxel and Sorafenib co-delivery	HepG2 (liver cancer)	2021	[133]
Biotin- PAMAM G4	Paclitaxel	A549 (lung cancer)	2019	[134]
PAMAM	Curcumin and siRNA	HeLa (cervical cancer)	2020	[135]

## 9 Magnetic nanoparticles (MNP)

Magnetic nanoparticles (MNP) have become increasingly popular among various drug delivery systems in recent years. Magnetic nanoparticles composed of cobalt and nickel have been investigated; however, iron oxide nanoparticles are the most extensively used in medical applications due to their low toxicity [136]. An MNP needs to have the following properties to be used in biological systems: i) high magnetization, ii) contrast properties for imaging, iii) surface coating to ensure stability, iv) optimized half-life and zeta-potential values, and v) ability to respond to an external stimulus [17]. Magnetic nanoparticles are usually coated with a layer such as a phospholipid [137], proteins [138], dendrimers [139], polysaccharides [140], or synthetic polymers [141] to ensure their stability in the body and provide a surface for functional group attachment.

### 9.1 Superparamagnetism

Several kinds of magnetism exist in nature such as diamagnetism, ferromagnetism, antiferromagnetism, ferrimagnetism, and paramagnetism. Among these, superparamagnetism is preferred for MNPs in biomedicine. Supermagnetism occurs in magnetic nanoparticles which are extremely small (<30 nm diameter). These small particles experience random flipping of magnetic moments due to the thermal fluctuations in a magnetic field. In the absence of a magnetic field, the average magnetization becomes zero due to the orientation of the magnetic moments [8]. The time between one-moment flip and the next is called the Néel relaxation time, portrayed by the Neel-Arrhenius relation as follows:

$$\tau =\tau_{0}\;\exp\left(KV/k_{B}T\right)$$

where $\tau_{0}$ is the time between flips, K is magnetic anisotropy energy density, V is the volume of the particle, $k_{B}$ is the Boltzmann constant and T is the temperature [142].

**Fig. 7:**
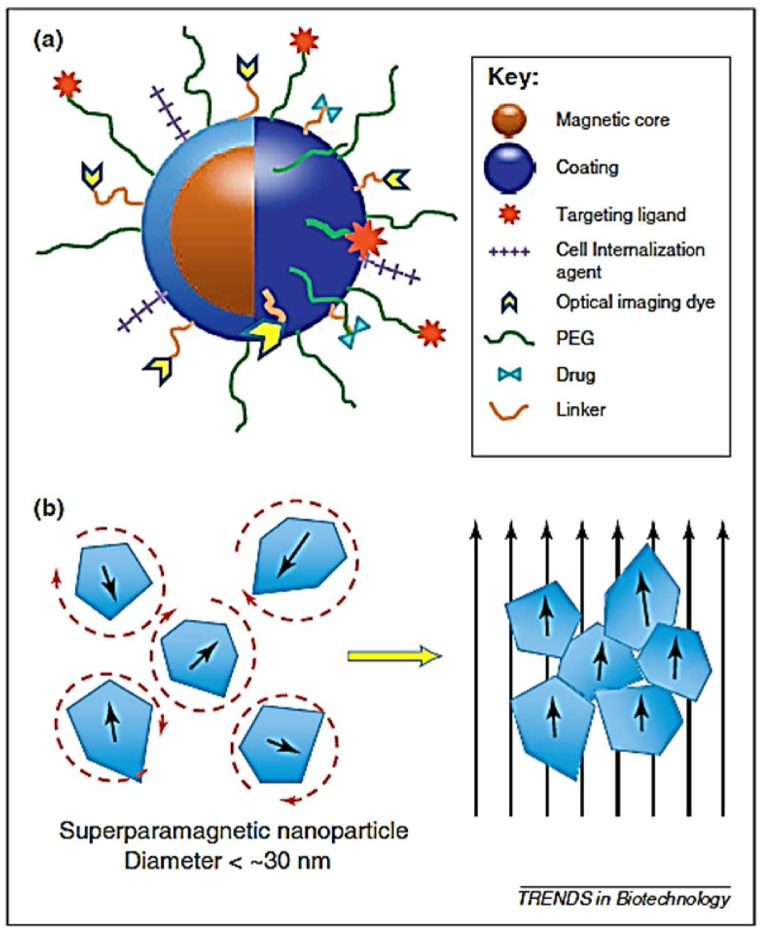
(a)Diagram of magnetic nanoparticle with multi-functional surface molecules. (b)Illustration of SPIONS response to a magnetic field. The circular dashed lines represent randomization in the absence of a magnetic field. Reprinted with permission from Ref. [8], Cole et al., “Cancer theranostics: the rise of targeted magnetic nanoparticles,” *Trends Biotechnol*, vol. 29, no. 7, pp. 323–332, Jul. **2011**.

Superparamagnetic iron oxide nanoparticles (SPIONs) offer various advantages, such as higher magnetic susceptibility and magnetic saturation in the presence of an external magnetic field compared to paramagnetic materials [143]. This increased magnetic susceptibility is due to the reorientation of individual Fe_3_O_4_ crystals in a magnetic field, as shown in Fig. 7 [8]. Fig. 7(a) shows a schematic of magnetic nanoparticle with multi-functional surface molecules, while Fig. 7(b) illustrates how SPIONS react in the presence of an external magnetic field. In nanomedicine, superparamagnetic nanoparticles are highly preferred due to their ability to avoid aggregation. If nanocarriers aggregate, they can promote their detectability by macrophages, resulting in failure of treatment [17]. To prevent particle agglomeration, magnetic nanoparticles are usually stabilized in a colloidal liquid solution known as ‘ferrofluids’ [130].

SPIONS exhibit a host of advantageous properties, such as the ability to modify the surface, intrinsic magnetic properties for MRI usage, and biocompatibility when coated with appropriate materials. Additionally, studies have shown that SPIONS are mechanically and chemically stable, have low polydispersity, predictable pharmacological properties and adequate drug release rate control [144]. A major disadvantage for other nanocarriers is biodegradability and elimination. This is not a concern for SPIONS since they are eliminated from the body through metabolic iron pathways [144].

### 9.2 Methods of SPION synthesis

There are numerous ways to synthesize SPIONS, depending on the application. They are usually classified into physical, biological, or chemical synthesis methods. Physical methods include laser-induced pyrolysis, pulsed laser ablation, gas-phase deposition, aerosol production, and electron beam lithography. Biological methods include using microbes such as fungi, bacteria, or proteins like ferritin. Lastly, chemical methods include co-precipitation, thermal decomposition, combustion synthesis, microemulsion, and sonochemical methods. These chemical methods are considered to provide the ideal size and composition of SPIONs, and hence, are the most commonly used [145].

One of the simplest and most commonly used SPION synthesis methods is the co-precipitation method [17]. This method allows ferrous and ferric salts to co-precipitate in an aqueous solution. Equation (1) represents one of the reactions used in this synthesis:

(Equation 1) $$2Fe^{3+}+Fe^{2+}+8OH^{-}\;\to \;Fe_{3}O_{4}+4H_{2}O$$

Several advantages exist in using this method, such as quick synthesis, high yield, and versatility. However, certain disadvantages exist, such as polydispersity, poor crystallization, and varying diameters [143].

For a more uniform size range, researchers prefer to use the thermal decomposition technique to synthesize SPIONs. This technique reacts organometallic precursors in organic solvents at temperatures above 200 °C. Earlier, using a hydrophobic coating such as oleic acid was a limitation in this process. However, with advancements in surface modification and using coatings that are stable at elevated temperatures, it is now possible to create coated SPIONs efficiently using the thermal decomposition method [146].

The hydrothermal technique requires a metal lineolate, water-ethanol solution and an ethanol-linoleic acid liquid at extreme temperature and pressure conditions. It is said that the ideal temperature would be 220 °C, and the ideal pressure would be above 10^7^ Pa for a 72-h reaction. The size of the synthesized particles would depend on the precursors, total reaction time, and temperature. The advantage of this hydrothermal technique is that it requires no post-treatments or organic solvents, making it an environment-friendly process [147].

Yet another commonly used method for SPION synthesis is the solution combustion method. This remarkably simple and rapid process involves a self-sustained reaction of oxidizer and fuel. The oxidizer used is usually a metal nitrate (iron nitrate, cobalt nitrate, etc.), while the fuel can be urea, glycine, hydrazides, etc. [148]. In recent years, several novel methods such as microwave-assisted synthesis [149], and sonochemical [150] methods have also been employed for SPION synthesis.

### 9.3 Surface coating for magnetic cores

To prevent iron leaching, aggregation and ensure long-term stability, the magnetic cores need to be coated with an appropriate material. The surface coating prevents oxidation and corrosion of the iron core, while increasing water solubility and stability. The main techniques for coating include post-synthesis grafting, post-synthesis adsorption, or *in situ* coating [144]. The most common approach is the *in-situ* coating approach, where the coating material is added to the reaction solution. Citrate-coated SPIONS have been reported in several studies [151,152] for use in the human body due to their biocompatibility. However, citrated MNPs can also coagulate at exceptionally low concentrations. Moreover, citric acid has the tendency to form complexes with the surface of the iron ions.

### 9.4 Applications of SPIONS in cancer drug delivery

In the past decade, increased attention has been placed on using SPIONs for various biomedical applications such as magnetic resonance imaging (MRI), positron emission tomography (PET), fluorescence imaging, regenerative medicine, biosensing and drug delivery [144]. More recently, magnetic nanoprobes have been employed for the early diagnosis of cancer. This is achieved through the unique fluorescent and magnetic properties of MNPs. SPIONs have also been exploited for their use as MRI imaging contrasts for molecular imaging.

Magnetic nanoparticles have also been used to treat cancer, through magnetic hyperthermia [153]. This technique involves accumulating the MNPs at the tumour site and subjecting them to an alternating current field. The MNPs absorb this energy and increase the internal tumour temperature to around 41 °C–47 °C, causing the cancer cells to die. An even more futuristic application of magnetic nanoparticles is in their use as magnetic nanorobots. These nanorobots have the capability to ‘drive’ or move around the bloodstream to deliver therapeutics to diseased cells. The nanorobots are synthesized in spiral or helical structures to achieve this purpose [154].

In one study, gelatine-coated iron oxide NPs were loaded siRNA for the treatment of colorectal cancer. The gelatine coating makes the carrier biocompatible and increases the storage stability [155]. Polyphenol-coated magnetic nanocarriers were also used to improve the synergetic effect when combined with immune/phototherapy, which showed excellent tumour growth inhibition and metastasis [156]. Table 9 outlines the recent studies of MNPs used to delivery chemotherapy drugs in cancer therapy.

**Table 9:** Summary of *in vivo* studies using magnetic nanoparticles for cancer drug delivery.

MNP type	Anti-cancer agent	Cell line for *in vivo* testing	Year study published	Reference
Albumin- Fe_3_O_4_	Doxorubicin	MCF-7 (breast cancer)	2019	[157]
Hyaluronic acid- Fe_3_O_4_	Methotrexate	A549 and CD44 (lung cancer)	2022	[158]
Folate - Fe_3_O_4_ NIPAAm-co-IA- Fe_3_O_4_	Doxorubicin	Tumor bearing mice	2022	[159]
Fe_3_O_4_ NPs with Au shell and pectin	Curcumin	HeLa (cervical cancer)	2021	[160]
Tannic acid - Fe_3_O_4_	Doxorubicin	HCT116 and LoVo (colon cancer)	2022	[161]
Tannic acid/SiO_2_ - Fe_3_O_4_	Doxorubicin and Methotrexate	MCF-7 (breast cancer)	2020	[162]
Fe_3_O_4_- mesoporous silica	Disulfiram	MCF-7 (breast cancer)	2021	[163]
Dextran - Fe_3_O_4_	Doxorubicin and Cetuximab	A549 (lung cancer)	2019	[164]

## 10 Mesoporous silica nanoparticles (MSNP)

**Fig. 8:**
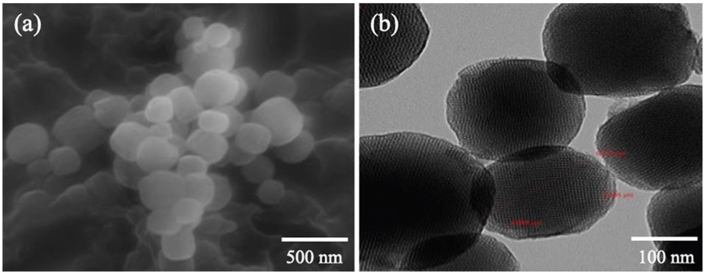
Mesoporous silica nanoparticles as seen under a) Scanning Electron Microscope (SEM), and b) Transmission Electron Microscope (TEM). Reprinted with permission from Ref. [166], Alfawaz et al., “Surface functionalization of mesoporous silica nanoparticles with Brønsted acids as a catalyst for esterification reaction,” *J. King Saud Univ. Sci*., vol. 34, no. 5, p. 102106, Jul. **2022**.

Mesoporous silica nanoparticles (MSNPs) are a relatively novel range of nanoparticles consisting of a honeycomb-like silica structure with many empty channels that can entrap molecules [19]. They were first discovered by Kresge and his co-workers in 1992. Their discovery was motivated by the desire to construct crystalline microporous zeolites with large pores which could accommodate larger molecules [165]. Fig. 8(a) and (b) illustrates the morphology of MSNPs using scanning and transmission electron microscopes, respectively [166]. Currently, one of the most advanced applications of MSNPs is in the field of nanomedicine. They have especially gained widespread attention as a potential nanocarrier candidate for anticancer drug delivery. The high stability and large surface areas of MSNPs offer excellent loading capacities for hydrophobic and hydrophilic drug molecules. Furthermore, they can also be modified with functional groups for targeted delivery to cancer cells [20].

### 10.1 Methods of mesoporous silica nanoparticles synthesis

Cai [167], Fowler [168], and Nooney [169] were the first to successfully synthesize and report mesoporous silica nanoparticles in 2001 using the ‘templating method.’ Over the past decade, researchers have extensively investigated the synthesis of MSNPs with varied dimensions, sizes, morphologies, and pore structures. MSNPs are fabricated by differing fabrication conditions, such as the pH of the reaction mixture, the characteristics of the surfactants, and the concentrations of silica. The templating method is by far the most commonly used technique to synthesize MSNPs [170].

One of the very first template methods used cationic surfactants, as demonstrated by Chao and co-workers [171]. They did this by quickly increasing the pH of the silica from 2.0 to the range of 6.0–9.0, to induce strong electrostatic forces between the cationic surfactant and silica. This further assisted in assembling the silica-surfactant particles whose size could be controlled by changing the reactant concentrations [171]. Hollow MSNPs created via soft templates have received widespread attention in recent years as they are known to improve loading capacity and encapsulation functionality. Microemulsion droplets [172], micelles [173], and vesicles [174] have been employed to create such hollow MSNPs.

Although the soft templating method for the synthesis of MSNPs is widely accepted, it comes with a few disadvantages, such as the co-existence of mixed structures and forms, lowered rigidity, and wider particle size distribution. These templates are easily removable after synthesis via simple methods such as acid-dissolution or calcination without destroying the actual MSNPs. MSNPs can also be synthesized without using a hard or a soft template through self-assembly. This technique is cost-effective and simple without needing additional surfactants [175]. In addition to these conventional templating methods, many novel methods have also been reported in the literature, such as aerosol-assisted assembly [176] and colloidal crystal templating [177].

### 10.2 Applications of mesoporous silica nanoparticles in cancer drug delivery

Mesoporous silica nanomaterials (MSNPs) have been thoroughly investigated as a potential nanomaterial for cancer therapy owing to their functionality, optical properties, high biocompatibility, ability to control size, and advanced drug encapsulation efficiency [178]. Vallet-Regi and colleagues were the first to report the use of MSNPs as a novel drug delivery system in 2001 [179].

Chrysin, a potential anti-cancer agent was tested with folic acid conjugated polyacrylic acid-capped MSNP for the treatment of breast cancer. Results indicated apoptosis through mitochondrial dysfunction was observed in the cancer cells [180]. MSNPs have also been employed for the combination treatment of chemotherapy and phototherapy [181]. A more interesting, recent study shows the synthesis of mesoporous biogenic silica nanoparticles from agricultural waste or wheat and rice husk. This was done by the acid leaching of silica from the husk, followed up sodium silicate addition via a sol-gel process to form the MSNPs. The MSNPs were loaded with DOX, and their efficiency was tested against MCF-7 and HFF-2 cancer cell lines [182]. Table 10 outlines the recent studies of MSNPs used to deliver chemotherapy drugs in cancer therapy.

**Table 10:** Summary of *in vivo* studies using mesoporous silica nanoparticles (MSNP) for cancer drug delivery.

MSNP type	Anti-cancer agent	Cell line for *in vivo* testing	Year study published	Reference
Folic acid – polyacrylic acid capped MSNP	Chrysin	MCF-7 (breast cancer)	2023	[180]
Polydopamine - MSNP	Gefitinib	AGS (gastric cancer)	2022	[183]
Lactobionic acid - MSNP	Doxorubicin	HepG2 (liver cancer)	2022	[181]
Zinc, amine, graphene oxide - MSNP	Gingerol and Letrozole	MCF-7 (breast cancer)	2022	[184]
Aptamer and peptide conjugated MSNP	Doxorubicin	MCF-7 (breast cancer)	2022	[185]
PBA/PAA - MSNP	Cuminaldehyde	MCF-7 (breast cancer)	2022	[186]
MSNP from agricultural waste	Doxorubicin	MCF-7 and HFF-2 (breast cancer)	2021	[182]
Glycosylated/chitosan MSNP	Capecitabine	HCT-116 (colon cancer)	2021	[187]
Folic acid - MSNP	Doxorubicin	ZR-75-1 and T47-D (breast cancer)	2021	[188]
Trastuzumab - MSNP	Doxorubicin	SKBR3 (HER2 positive) (breast cancer)	2021	[189]
MSNP	Alamandine	4T1 (breast cancer) and A549 (lung cancer)	2021	[190]
Hyaluronic acid - MSNP	Curcumin	MCF-7 and MDA-MB-231 (breast cancer)	2021	[191]
Lactoferrin - MSNP	Pemetrexed	MCF-7 (breast cancer)	2020	[192]

## 11 Gold nanoparticles (AuNP)

Gold (atomic number 79) is a metal that is characterized by its bright yellow color with soft and ductile nature and low reactivity. Due to its rarity, resistance to corrosion and unique properties, gold has an excellent value. Interestingly, gold has been used in medical applications in early Chinese, Arabic, and Indian civilizations for the treatment of various diseases. More recently, there has been a growing interest in using gold nanoparticles in biological applications due to their low toxicity, stability and ease of synthesis [193]. Unlike bulk gold, the properties of its nanoparticles are quite different. For example, the color of AuNPs can be brown, purple, blue, orange, or red, depending on particle size. The melting point of AuNPs depends on their particle size. The size of the particle and the melting are related. This is due to the weakened attractive forces of the core due to a lowered number of neighboring atoms. This causes the surface atoms to gain energy, leading to a decrease in the melting point [194]. One of the earliest uses of gold nanoparticles in medicine was to treat rheumatoid arthritis [195]. Currently, AuNPs are being studied for their use as imaging agents, drug delivery vehicles and absorptive heating agents.

### 11.1 Methods of gold nanoparticles synthesis

**Fig. 9:**
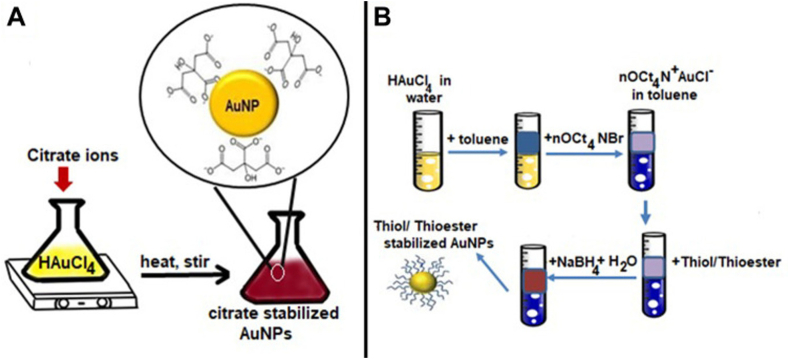
a) Turkevich method for AuNP synthesis, b) Brust method for AuNP synthesis. Reprinted with permission from Ref. [21], Amina et al., “A Review on the Synthesis and Functionalization of Gold Nanoparticles as a Drug Delivery Vehicle,” *IJN*, vol. 15, pp. 9823–9857, Dec. **2020**

The earliest method of spherical AuNP synthesis is referred to as the “Turkevich method” and was introduced in 1951. The basic principle in this method involves reducing Au^3+^ ions to Au^0^ atoms using reducing agents such as amino acids, citrate, or ascorbic acid. The resultant AuNPs are in the range of 1–2 nm. This limitation of size range encouraged researchers to discover other synthesis methods for a broader NP size range. Another method of synthesis was introduced by Brust et al. in 1994. It comprises a two-phase reaction to formulate AuNPs using an organic solvent. Tetraoctylammoniumbromide is usually used to transfer gold from the aqueous solution to the organic solvent. This is followed by using a reducing agent to reduce the gold to its atomic state [196]. Schematic diagrams of the Turkevich method and Brust method are illustrated in Fig. 9 (a) and (b), respectively [21].

The above-mentioned methods are used to fabricate spherical gold nanoparticles. However, to produce rod-shaped AuNPs, a seed-mediated growth technique is used. The first step of this synthesis involves preparing seed particles via reduction methods. This is followed by transferring the particles to a metal salt to prevent further nucleation [197]. Spherical- and rod-shaped gold nanoparticles vary in their optical properties, especially in terms of their LSPR. The LSPR or the ‘localized surface plasmon resonance’ is the oscillation of free electrons in the atom in the presence of an electric field. Gold spheres tend to exhibit only a single LSPR, while rod-shaped AuNPs exhibit two LSPRs [197].

### 11.2 Applications of gold nanoparticles in anticancer drug delivery

Gold nanoparticles can be utilized in cancer treatment for two modalities of treatment: induced hyperthermia or drug delivery. AuNPs can easily absorb near-infrared (NIR) light, increasing tumors' temperature, leading to hyperthermia and subsequent cell death [198]. Gold nanoparticles modified using polysaccharides or glycopolymers are used extensively in cell imaging and therapeutics. The glycopolymers, which are hydrophilic, enhance the dispersion properties and biocompatibility [199].

A comparative study was conducted by Vemuri and colleagues using biosynthesized AUNPs (b-AuNP) and loaded with quercetin, curcumin (CUR), turmeric, and paclitaxel (PTX). The cytotoxicity of these four individual formulations and their combination were tested in two breast cancer cell lines: MCF-7 and MDA-MB 231. The highest therapeutic efficiency was observed when a combination of all four b-AuNPs was used, revealing a synergistic effect [200]. Another study investigated the use of Hesperidin in combination with AuNPs. Hesperidin is a flavonoid glycoside with known anticancer activities. However, it is not used clinically due to poor solubility, low absorption, and low bioavailability. An *in vivo* study was conducted by injecting the Hsp-AuNPs into mice and measuring kidney and liver function markers. Histological images of major organs revealed no abnormalities of damage after injection of these novel nanoparticles [201].

In recent times, a more sustainable or ‘green’ method of Au NP synthesis from plants have been investigated in detail. These biosynthesized particles exhibit anticancer properties, especially against cervical cancer cell lines [202]. Moreover, scientists have also tried to combine chemotherapy with gene therapy using Au NPS to co-deliver siRNA and anti-cancer drugs. Results revealed Bcl-2 expression was inhibited with this multifunctional drug delivery system [203]. Table 11 outlines the recent studies of AuNPs used to deliver chemotherapy drugs in cancer therapy.

**Table 11:** Summary of *in vivo* studies using gold nanoparticles for cancer drug delivery.

Au NP type	Anti-cancer agent	Cell line for *in vivo* testing	Year study published	Reference
EGF – Au NP	Luteolin	MDA – MB – 231 (breast cancer)	2022	[204]
Au NP (biosynthesized)	Quercetin, Curcumin, Turmeric, Paclitaxel	MCF-7 and MDA-MB 231 (breast cancer)	2019	[200]
Au NP	Hesperidin	MDA-MB 231 (breast cancer)	2020	[201]
Au NP (biosynthesized)	Naringenin	PC3 (bone cancer)	2023	[205]
AuNP	Doxorubicin and siRNA	MCF-7 (breast cancer)	2022	[203]
Gelatin - AuNP	Methotrexate	MCF-7 (breast cancer)	2021	[206]
Gum acacia – Au NP	Gemcitabine	MDA – MB – 231 (breast cancer)	2020	[207]
AuNP@ collagen	5-fluorouracil	HeLa (cervical cancer) and A549 (lung cancer)	2022	[208]
AuNP	Docetaxel	H520 (lung cancer)	2019	[209]

## 12 Carbon nanotubes (CNT)

Ijima was the first to design carbon nanotubes (CNT) in 1991 [210] and have since driven scientists to use them in various application from energy to biomedicine. CNTs are graphite sheets rolled up into a tubular structure at the nanoscale. They can be two main types: a single graphene sheet rolled over called single-wall carbon nanotubes (SWCNTs), while additional graphene sheets rolled over a SWCNT result in a multi-wall carbon nanotube (MWCNT). The diameters and lengths can vary depending on the application; however, each end is usually capped by a fullerene structure [211].

Their unique structure gives them extraordinary mechanical, electrical and optical properties such as high elastic modulus (>1 TPa) and high strength [212]. CNT is known to possess at least twice the thermal conductivity of diamonds. They can withstand extreme temperatures of 750 °C and 2800 °C at vacuum and atmospheric pressures [213]. The thermal conductivity of SWCNT at room temperature is greater than 200 W/mK (bulk) and over 3000 W/mK for MWCNT (single). Another amazing characteristic of CNTs is their elasticity. They can be bent and twisted without damage on exposure to axial compressive forces [23].

In recent years, carbon nanosheets in the form of graphene oxide sheets have been investigated for drug delivery to cancer cells. In a study by Xu et al., graphene oxide sheets were conjugated to the anticancer drug Paclitaxel (PTX) via aminated polyethylene glycol chains. These novel nanocarriers were tested for the efficiency on a melanoma cancer-bearing mice model. Results revealed prolonged circulation time in the bloodstream compared to the drug alone. Additionally, the nanocarriers exhibited excellent tumor-targeting mechanisms, thus making carbon nanosheets an ideal carrier for drug delivery [214].

### 12.1 Advantages and disadvantages of carbon nanotubes

There are several advantages of CNTs. Their superior mechanical, magnetic, thermal, optical, and electrical properties make them highly desirable for nanotechnology applications. They exhibit rigidity and sturdiness owing to their tubular graphitic structure. They can carry electric current 1000 times higher than that of copper wire. CNTs have been exploited for use in many modern-day devices, such as scanning probe microscopes, microelectronic devices and many others [211].

However, with all these advantages, there are certain limitations too. The chemical inertness and hydrophobic nature of CNTs suggest that suspending them in a solution is difficult. In order to make them more soluble, CNTs can be modified with surfactants such as polyethylene glycol, and sodium dodecyl sulphate. Moreover, CNTs tend to aggregate, rendering them ineffective. To overcome this problem, their surface can be modified with covalent or non-covalent ligands [215].

### 12.2 Methods of carbon nanotubes synthesis

The synthesis of CNTs involves many parameters such as temperature, pressure, reactor geometry, catalyst, etc. Ijima, in 1991, demonstrated the use of the ‘arc discharge’ method to synthesize the very first carbon nanotubes [210]. Since then, researchers have developed various synthesis methods. The commonly used synthesis methods include laser ablation, arc discharge, chemical vapor deposition (CVD), and plasma-enhanced CVD.

The arc discharge or arc evaporation technique was first used in 1985 to synthesize Buckminster fullerenes [216]. It is a relatively easy procedure where a gas is dissociated electrically to generate plasma to produce SWCNT and MWCNT. Two graphite electrodes, of which the anode was filled with catalyst and carbon precursor, are used in this method. The two electrodes were heated to a high temperature and a DC current of 50–100 A was provided to produce CNTs [217].

In the laser ablation technique, a laser beam (continuous or pulsed) is used to vaporize a graphite element in a furnace at 1200 °C and an inert gas is introduced to maintain pressure at 500 Torr [218]. This method was first used by Smalley et al. in 1995 to produce SWCNTs. The limitation of this technique is that a number of properties affect its synthesis, such as chemical and structural composition, temperature, laser properties, oscillation wavelength, etc. [219].

Nowadays, chemical vapor deposition (CVD) is commonly used because of its simplicity, inexpensive procedure, and low temperature/pressure requirement. This method also produces CNTs with higher yield and purity compared to the above-mentioned methods. In this technique, a tubular reactor with a metal (Co, Ni, or Fe) catalyst is used through which a hydrocarbon vapor passes at a high temperature (600–1200 °C). This causes the hydrocarbon to decompose, and the CNT gets deposited on the catalyst [220]. More recently, plasma-enhanced chemical vapor deposition (PECVD) has been investigated to produce CNTs. It is an alternative at lower temperatures (i.e., room temperatures) compared to the extremely high temperatures in the conventional CVD processes [221].

### 12.3 Applications of carbon nanotubes in anticancer drug delivery

**Fig. 10:**
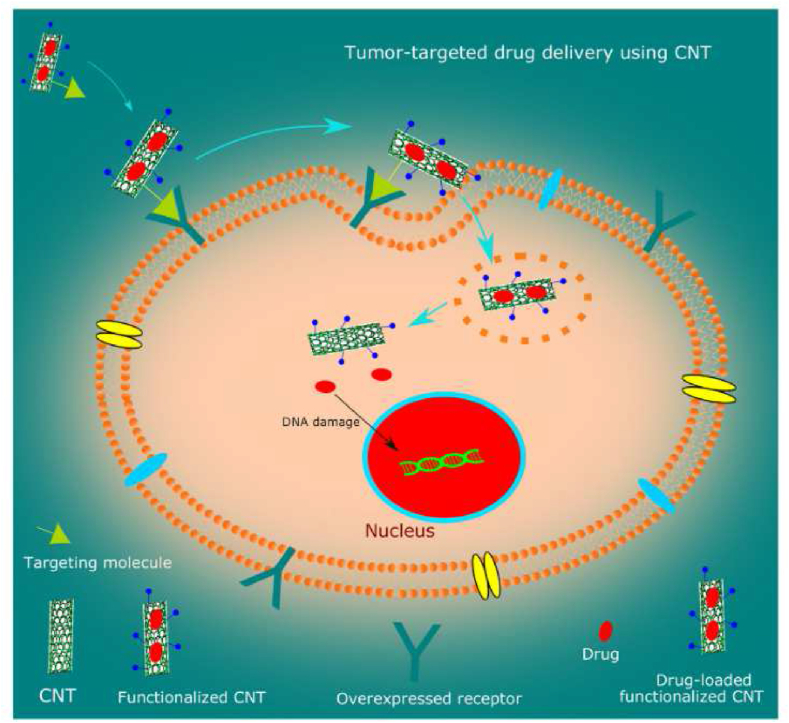
Illustration of tumor-targeted drug delivery using carbon nanotubes. Reprinted with permission from Ref. [222], Raphey et al., “Advanced biomedical applications of carbon nanotube,” *Mater. Sci. Eng. C*, vol. 100, pp. 616–630, Jul. **2019**.

Certain favorable properties of CNTs such as high loading capacity, surface area, and stability make them ideal candidates for drug delivery to cancer cells. Fig. 10 illustrates how drug-loaded carbon nanotubes effectively deliver anti-cancer drugs to tumors [222]. In modern times, supramolecular organic nanotubes have been investigated for the potential in cancer drug delivery. These nanocarriers self-assemble via non-covalent bonds such as metal, amphiphilic, hydrogen or van der Waals forces. These nanocarriers are soft matter, with distinct organic channels. Drug loading is carried out through chemical boning or physical encapsulation [223].

Nanotubes made of materials other than carbon have also piqued researchers’ interest. One study investigated the cell viability of titanium oxide nanotubes, loaded with the antibiotic drug Ciprofloxacin. PLGA was coated on the nanotube to slow down the drug release rate [224]. In another study, boron nitride nanotubes were used to deliver Thioguanine to cancer cells [225]. Although many studies have been conducted to understand the diverse applications of CNTs in cancer drug delivery, their use in clinical settings still remains restricted. This is primarily due to their prominent levels of toxicity and low biocompatibility. CNTs need to be surface-modified with appropriate molecules before they can enter the bloodstream to deliver chemotherapeutics. Table 12 outlines the recent studies of CNTs used to deliver chemotherapy drugs in cancer therapy.

**Table 12:** Summary of *in vivo* studies using carbon nanotubes for cancer drug delivery.

CNT type	Anti-cancer agent	Year study published	Reference
SWCNT	Doxorubicin and Camptothecin	2023	[226]
Poly(L-histadine) CNT	Doxorubicin	2022	[227]
Chitosan and PEG coated CNT	Doxorubicin and Imatinib	2022	[228]
poly(N-isopropylacrylamide-block-poly(2-(4-formylbenzoyloxy) ethyl methacrylate) - CNT	Doxorubicin	2019	[229]
CNT (Fe)/hydroxyapatite	Doxorubicin	2019	[230]
NC_3_ CNT	Fluorouracil	2022	[231]
BC_3_ CNT	Thiotepa	2022	[232]
MWCNT	5-Fluorouracil	2021	[233]

## 13 Quantum dots (QD)

**Fig. 11:**
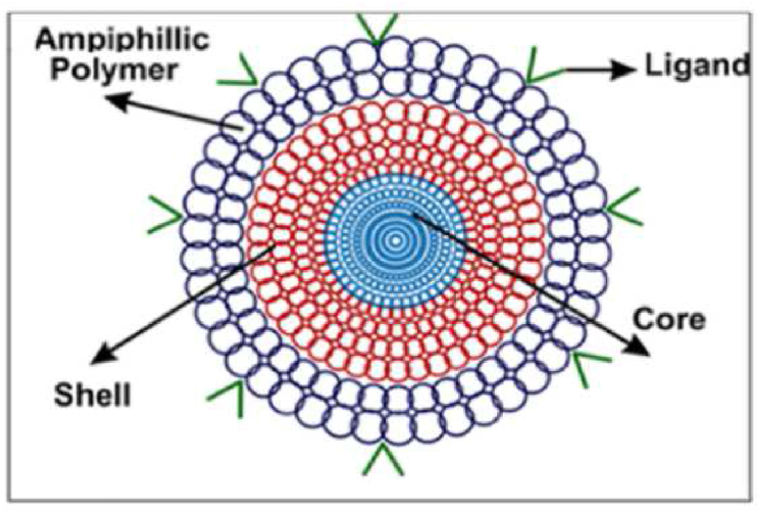
Structure of semiconductor quantum dot. Reprinted with permission from Ref. [234], Gidwani et al., “Quantum dots: Prospectives, toxicity, advances and applications,” *J Drug Deliv Sci Technol*, vol. 61, p. 102308, Feb. **2021**.

Quantum dots (QD) are fluorescent semiconductor-based nanocrystals that have recently been extensively explored for their unique features and use in a variety of industries. They are usually composed of heavy metal or inorganic materials ranging from 2 to 10 nm in size. The structure of QDs includes a semiconductor core, cap, and shell. The cap is responsible for providing solubility in solutions, while the shell protects the core and can be used to attach targeting ligands [234]. A structure of a semiconductor quantum dot is illustrated in Fig. 11 [234]. The fluorescence in QDs is a result of the bandwidth gap between valence and conducting electrons. When a photon of higher energy than the spectral band of the core gets absorbed, an electron gets excited to the conduction band, resulting in excitation of the electron and consequent fluorescence.

QDs have many unique properties that make them ideal for various applications. QDs with a broad excitation spectrum can be excited by a single light source. They can be easily detected due to the ‘Stokes shift,’ which is the difference between the absorption and emission wavelengths of QDs. Additionally, they have a longer luminescence lifetime due to their inorganic distribution and higher emission compared to other organic dyes. It is estimated that the brightness of QDs is at least 10 to 20 times greater than the brightness emitted by single fluorophore molecules [235].

Despite these advantages, several disadvantages also exist in using QDs in medicinal applications. Cadmium and selenium QDs can deposit in the bronchi and cause acute and chronic issues. Moreover, there are specific environmental concerns with these metals. They can deposit and stay in the environment without degradation for at least 15–20 years. The toxicity of these nanoparticles needs to be further investigated before they can be safely used in any application, especially in the biomedical field. The toxicity depends on various factors such as concentration, charge, size, and mechanical stability [236].

### 13.1 Methods of quantum dots synthesis

The two main metal precursors used in QD synthesis are tellurium or cadmium, which form 2–10 nm cores in diameter [235]. The most common synthesis methods for QDs are either top-down processing methods or bottom-up approaches. Techniques in this method include X-ray lithography, molecular beam epitaxy (MBE), ion implantation, and e-beam lithography. Alternatively, in the bottom-up approach, nanoparticles are synthesized using smaller structures such as atoms, molecules, or crystals. These include preparing colloidal QDs through self-assembly [237]. For top-down synthesis, a bulk semiconductor material is used to achieve QDs of 20–40 nm diameter. This is achieved through advanced techniques such as electron beam lithography, reactive-ion etching, or wet chemical etching [238].

Both the top-down processing method and bottom-up processing methods have their own pros and cons. Top-down approaches are simpler and easier to execute; however, they cause surface irregularities and imperfections. On the other hand, the bottom-up approach yields nanoparticles with fewer surface defects and homogenous chemical composition and is, hence, more widely used in commercial applications [239].

### 13.2 Applications of quantum dots in anticancer drug delivery

QDs are the perfect candidate for diagnostics and biomedical applications because of their biocompatibility, narrow emission wavelength, lowered toxicity, and high luminescence [240]. Currently, studies on using QDs in cancer drug delivery are limited to cells and small animals due to the absence of knowledge about *in vivo* degradation and toxicity of these materials.

**Fig. 12:**
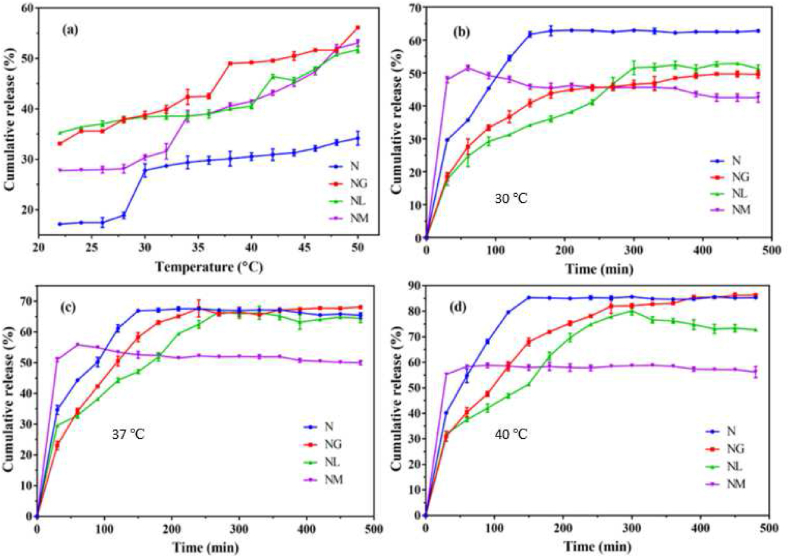
Cumulative percentage release of drug from nanohydrogels a) at varying temperatures, b) at 30 °C, c) at 37 °C, and d) at 40 °C. Reprinted with permission from Ref. [241], Havanur et al., “Poly (N,*N*-diethyl acrylamide)/functionalized graphene quantum dots hydrogels loaded with doxorubicin as a nano-drug carrier for metastatic lung cancer in mice,” *Mater. Sci. Eng. C*, vol. 105, p. 110094, Dec. **2019**.

In one study, graphene quantum-dots-incorporated hydrogels were used as a drug carrier to treat melanoma. The synthesized hydrogels were 68.1–87.5 nm in diameter, and doxorubicin (DOX), an anticancer drug, was incorporated into the nanocarriers. Excellent release of the drug from the nanohydrogels was observed, and the cumulative drug release profile was found to follow the Fickian diffusion release mechanism. The drug release was higher at elevated temperatures, as seen in Fig. 12. *In vivo* studies were conducted on mice models to study the efficiency and cytotoxicity of the nanoparticles [241].

In addition to cancer drug delivery, quantum dots have also been investigated to deliver drugs to other diseased cells. For instance, heteroatom functionalized quantum dots were synthesized for the drug delivery of isoniazid to cure tuberculosis [242]. Another novel class of QD is cadmium telluride (CdTe) QD which is beneficial due to its large bandwidth and binding energy. It has been used in various biomedical applications as an anti-fungal, antibacterial, antioxidant, etc. The effects of using CdTe QD as drug nanocarriers are still unclear due to their potential toxicity [243].

Carbon quantum dots (CQD) are a relatively new member of the carbon family which displays carbogenic fluorescent properties. A carboxyl group is present on CQDs which contribute to increased solubility, biocompatibility, photostability and excellent fluorescence property [244]. Graphene quantum dots have also been investigated for their use as anti-cancer drug carriers. Gautam et al. [245] and Prabakar et al. [246] used graphene QDs to deliver Gefitinib and B-Lapachone anticancer agents, respectively. Both studies confirmed the successful conjugation of the drug into the QD and excellent release rate *in vivo.* Table 13 outlines the recent studies of quantum dots used to deliver chemotherapy drugs in cancer therapy.

**Table 13:** Summary of *in vivo* studies using quantum dots for cancer drug delivery.

QD type	Anti-cancer agent	Cell line for *in vivo* testing	Year study published	Reference
PEG conjugated graphene QD	Gefitinib	NCI–H522 (lung cancer)	2022	[245]
Graphene QD	B-Lapachone	MCF-7 (breast cancer)	2022	[246]
Folic acid conjugated graphene QD	Curcumin	MCF-7 (breast cancer) and MG-63 (bone cancer)	2023	[247]
ZnO QD	Doxorubicin	MCF-7 (breast cancer)	2022	[248]
CdSe/ZnS QD	10-hydroxycamptothecin	HeLa (cervical cancer)	2022	[249]
Quinic acid conjugated carbon QD	Gemcitabine	MCF-7 (breast cancer)	2021	[250]
Chitosan/aptamer carbon QD	5-fluorouracil	MCF-7 (breast cancer)	2020	[251]
QD crosslinked carboxymethyl cellulose hydrogel	Doxorubicin	HT29 (colorectal cancer)	2020	[252]
Chitosan – CdS QD	Sesamol	MCF-7 (breast cancer)	2019	[253]
ZnFe_2_O_4_@SiO_2_ graphene QD	Doxorubicin	HeLa (cervical cancer)	2022	[254]
Transferrin- carbon QD	Doxorubicin	MCF-7 (breast cancer)	2021	[255]

## 14 Future and challenges of anticancer nanomedicine

The collective urge to treat cancer and develop effective treatment strategies has made cancer nanomedicine one of the most promising therapeutic platforms of the future. This field of targeted drug delivery and cancer nanomedicine will definitely remain an exciting area for future developmental research. The commercialization of nanotechnology-related health products has been increasing worldwide [256]. Currently, Europe and North America are leading in commercial nanomedicine products, owing to their powerful regulatory frameworks [257]. However, Asia is also rapidly expanding its nanomedicine research and may become a leading scientific contributor in the near future. Clinical translation remains challenging and stronger regulations are required to introduce nanomedical products in the Asian market.

Although the use of nanomedicine for cancer therapy sounds promising and exciting, it comes with a few evident challenges. Firstly, the majority of the studies are based on exploiting the EPR effect to deliver anti-cancer drugs to tumor cells. In reality, however, the EPR effect is not as simple as it sounds. It involves many subsequent steps such as *in vivo* blood circulation, drug penetration, accumulation, and drug release. Moreover, diverse types of tumors portray different anatomy which may not adhere to the EPR effect. There is also some evidence to suggest that the EPR effect is a mere hypothesis, and that nanocarriers internalize into cells via *trans*-endothelial pathways [258].

Secondly, the toxicity and long-term physiological implications of nanomaterials in the bloodstream has not yet been fully understood. For example, Doxil was one of the first FDA-approved liposomal doxorubicin formulation for cancer treatments. It was declared safe with low toxicity and myelosuppression values. However, recent research suggests new side effects in patients administered with Doxil, including hypersensitivity and hand-foot syndrome [259].

Thirdly, to commercialize these technologies and make them clinically available, specific parameters need further investigation. Large-scale manufacturing may be a potential challenge hindering the development of many nanomedicine-based anticancer therapeutics. Most of the nanomedicine studies found in literature are conducted on a lab-scale basis, and it is unclear how such technologies would be scaled up. Many nanotherapeutics fail to be made commercially available because of their inability to be produced at a large scale [257]. Scaling up and mass production of nanotherapeutics is expensive and difficult and may not correlate in the final characteristics of the formulation with those produced at a smaller scale in the lab. Therefore, it is better to carefully analyze the cost-benefit of the nanoparticles early in the nanodrug development phase. An example of potential commercial nanotechnology was introduced by Khan and his colleagues in the form of ethanol-based proliposome tablets as an alternative to lab-produced liposomes [260].

Finally, another challenge in commercializing this technology is the lack of regulatory rules and standards for these therapeutics' safe manufacture and quality control. The Food and Drug Administration (FDA) and the European Medicines Agency (EMA) only have guidance documents to regulate nanotherapeutic products. As a result, there are no legal procedures in place for these products' production and clinical use. This is a major roadblock to overcome at the earliest to ensure that nanomedicine is available to cancer patients clinically [261].

## 15 Conclusions

Traditional modalities of treatment, especially chemotherapy, cause many side effects to the patients. With the development of nanotechnology and targeted drug delivery systems, these side effects could be minimized; thus, increasing the positive outcome of the cancer treatment. Each nanoparticle type introduced in this review offers its own pros and cons of effectively delivering anti-cancer drugs. Currently, a great deal of research is being done on discovering and manipulating nanoparticles for drug delivery, as mentioned in the review. Nanomedicine is still in its infancy and will require great collaborative efforts to translate it into a clinical setting for cancer patients. More in-depth studies are required to maximize the efficiency of these nanocarriers, by increasing safety, biocompatibility, and bioavailability. It is also imperative to introduce regulatory frameworks and processes for the smooth clinical translation of these products. The field of nanomedicine for cancer is continually making progress and significant advancements, and there is no doubt that using nanoparticle-based technology will be the future of cancer therapeutics.

## Declaration of competing interest

The authors declare that they have no known competing financial interests or personal relationships that could have appeared to influence the work reported in this paper.

Acknowledgement

Open Access funding provided by the Qatar National Library
